# Meta-analysis of association between TCF7L2 polymorphism rs7903146 and type 2 diabetes mellitus

**DOI:** 10.1186/s12881-018-0553-5

**Published:** 2018-03-07

**Authors:** Weiyue Ding, Li Xu, Lejun Zhang, Zhijie Han, Qinghua Jiang, Zhe Wang, Shuilin Jin

**Affiliations:** 1 College of Computer Science and Technology, Harbin Engineering University, No.145 Nantong Street, Nangang District, Harbin, 150001 China; 2 School of Information Engineering, Yangzhou University, No.196, Huayang West Road, Yangzhou, 225127 China; 3 School of Life Science and Technology, Harbin Institute of Technology, No.92 Xidazhi Street, Nangang District, Harbin, 150001 China; 4 Key Laboratory of Symbolic Computation and Knowledge Engineering of Ministry of Education, Jilin University, No.2699, Qianjin Avenue, Qianweinan District, Changchun, 130012 China; 5 Department of Mathematics, Harbin Institute of Technology, No.92, Xidazhi Street, Nangang District, Harbin, 150001 China

**Keywords:** T2DM, Polymorphism, rs7903146, Meta-analysis

## Abstract

**Background:**

Large scale association studies have found a significant association between type 2 diabetes mellitus (T2DM) and transcription factor 7-like 2 (TCF7L2) polymorphism rs7903146. However, the quality of data varies greatly, as the studies report inconsistent results in different populations. Hence, we perform this meta-analysis to give a more convincing result.

**Methods:**

The articles, published from January 1st, 2000 to April 1st, 2017, were identified by searching in PubMed and Google Scholar. A total of 56628 participants (34232 cases and 22396 controls) were included in the meta-analysis. A total of 28 studies were divided into 4 subgroups: Caucasian (10 studies), East Asian (5 studies), South Asian (5 studies) and Others (8 studies). All the data analyses were analyzed by the R package meta.

**Results:**

The significant association was observed by using the dominant model (OR = 1.41, CI = 1.36 - 1.47, *p* < 0.0001), recessive model (OR = 1.58, CI = 1.48 - 1.69, *p* < 0.0001), additive model(CT vs CC) (OR = 1.34, CI = 1.28-1.39, *p* < 0.0001), additive model(TT vs CC) (OR = 1.81, CI = 1.69-1.94, *p* < 0.0001)and allele model (OR = 1.35, CI = 1.31-1.39, *p* < 0.0001).

**Conclusion:**

The meta-analysis suggested that rs7903146 was significantly associated with T2DM in Caucasian, East Asian, South Asian and other ethnicities.

**Electronic supplementary material:**

The online version of this article (10.1186/s12881-018-0553-5) contains supplementary material, which is available to authorized users.

## Background

Diabetes is one of the largest global health emergencies in the twenty-first century. According to the International Diabetes Federation (IDF) [1], 46.5% of the adults with diabetes are undiagnosed, and 1 in 11 adults, about 415 million people, have diabetes. Every 6 s a person dies of diabetes (5.0 million deaths per year). By 2040, 1 in 10 adults, approximately 642 million people, will have diabetes. Notably, 12% of the global health expenditure, up to $673 billion, is dedicated to diabetes treatments, and the related take up most of the total expenditure.

The most prevalent form of diabetes is type 2 diabetes mellitus (T2DM), and in the developed countries up to 91% of the adults, who are being troubled by the diabetes, have T2DM. Excess body weight, physical inactivity, poor nutrition, genetics, family history of diabetes, past history of gestational diabetes and older age are risk factors that increase the rate of T2DM. Besides, T2DM is a complex disease, and and the function of the glycosylation plays a significant role [2, 3].

The SNP rs7903146(C/T) is a common variant in the gene TCF7L2, and allele T is the risk allele related to T2DM. The gene TCF7L2 is a transcription factor involved in the Wnt signaling pathway, and acts as a critical component of Wnt signalling and action [4–6]. The TCF7L2 gene product, a high mobility group box-containing transcription factor previously implicated in blood glucose homeostasis, is considered to act through the regulation of proglucagon gene expression in enteroendocrine cells via the Wnt signaling pathway [7]. In human islets, TCF7L2 expression associates positively with insulin gene expression [8, 9].

To address the genetic variations of T2DM, many scholars devoted themselves to the related research [10–16]. The common Pro12Ala polymorphism rs1801282 in PPAR *γ*, the E23K variant rs5219 in KCNJ11, the polymorphism of the 5-HT2C receptor rs3813929 and the VKORC1 polymorphism rs9923231 were found to be associated with T2DM [17–20]. In 2006, Grant SF, et al. [7] confirmed a strongly significant association between susceptibility related to T2DM and common variants in transcription factor 7-like 2 (TCF7L2) in Icelandic subjects, and the result was the same with case-control method in Danish cohort and U.S. cohort. In 2006, Cauchi et al. [21] reported that the T-allele of the single nucleotide polymorphism (SNP) rs7903146 increased the risk of T2DM in the French population with 2367 cases and 2499 controls.The same results were shown by Horikoshi, Yu and Barra in case of the Japanese population, African American population and Brasilia [22–24]. However, Zheng et al. [25] found no association between rs7903146 and T2DM in the Chinese population.

The quality of the data varies greatly, is one of the reasons that the studies report inconsistent results, and the small sample size is another reason. The statistical efficiency can be improved after combining some samples together. The collected data in the control group was tested by the Hardy-Weinberg Equilibrium (HWE) in view of the quality of data. Therefore, we conducted a meta-analysis of published studies involving rs7903146 and T2DM to achieve a more comprehensive result. Finally, a total of 28 studies from 26 single studies [4, 22–46] were collected to reevaluate the association between rs7903146 and T2DM.

## Methods

### Search strategy

The articles, published from January 1st, 2000 to April 1st, 2017, were identified by searching the keywords “rs7903146” and “type 2 diabetes mellitus” in PubMed and Google Scholar. The selected articles were written in English.

### Study selection criteria

We selected studies according to the following criteria: (1) The study was designed based on the case-control method. (2) The study evaluated the association between rs7903146 and T2DM. (3) The number of genotypes in case-controls groups was provided for calculating Odds Ratios (ORs). (4) The control group meets HWE. Besides, the *p* value of HWE was calculated by R program HWE version 1.2 [47]. If *p* < 0.05, the article was preserved, otherwise the article was removed.

### Data extraction

We extracted the following information from each study: (1) the first author of each article; (2) the publication year of each article; (3) the population of the study; (4) the ethnicity of individuals in each study; (5) the number of the rs7903146 genotypes both in cases and controls; (6) *p* value of HWE in the control group. We used R package meta to analyze the data. We also referred to some other methods [48–51] to conduct the meta-analysis.

### Choice of genetic model

The rs7903146 has two alleles: C and T. We analyzed the association between rs7903146 and T2DM by using the dominant model (TT+CT versus CC), recessive model (TT versus CC+CT), additive model (CT versus CC), additive model(TT versus CC) and allele model (T versus C), respectively [52].

### Heterogeneity test

Odds Ratios and 95% confidence intervals (CIs) were calculated to assess the association between rs7903146 and T2DM. The two quantities, Cochran’s Q and I^2^, were adopted to evaluate the heterogeneity in different kinds of ethnic groups. Q approximately follows a chi square distribution with k-1 degrees of freedom (where k is the number of studies), and the *p* value can be used to measure the significance level of the heterogeneity. The value of I^2^, ranging from 0 to 100%, is calculated according to the formula, which is I ^2^ = (Q-(K-1))/Q*100%. The low, moderate, and high heterogeneity were labelled by I^2^ levels of 25%, 50% and 75%, respectively. If I^2^ is less than 50%, or p is more than 0.10, the fixed effect model is used, otherwise the random effect model is adopted.

### Meta-analysis and subgroup analysis

After the heterogeneity test, we used the R package meta to perform the experiment with the fixed effect model [53].

### Publication bias analysis and sensitivity analysis

Begg’s test [54] and Egger’s test [55] were selected for testing the publication bias. When a two-tailed value is less than 0.05, the publication bias is significant.

## Results

### Literature search

**Fig. 1:**
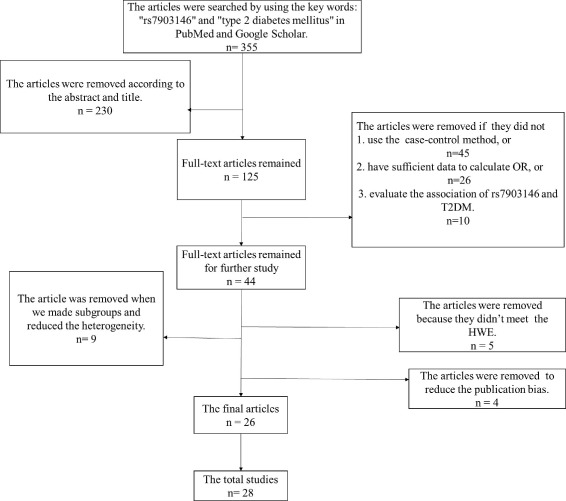
The flow chart of collecting articles for analyzing the association. And a total of 355 articles were identified by the search strategy. Firstly, a total of 230 articles were removed according to the title and abstract, and 45 articles were removed as the studies did not use case-control method, and 26 articles were removed as the studies did not have sufficient data to calculate OR, and 10 articles were excluded as they did not evaluate the association between rs7903146 and T2DM. After that 44 articles remained. Then, 5 articles were excluded as the control groups didn’t meet the Hardy-Weinberg Equilibrium (HWE), 9 articles were excluded when we made subgroup analyses and reduced the heterogeneity, and 4 articles were excluded as some LADA or type 1 diabetes patients were included in the case groups. Finally 28 studies from 26 articles were left

A flow diagram for the study selection process was shown in Fig. 1. A total of 355 articles were identified by the search strategy, abd 28 studies from 26 articles were left. The detailed information about the search strategy was displayed in Additional file 1: Table S1.

### Study characteristics

As shown in Table 1, a total of 56628 participants (34232 cases and 22396 controls) of 28 studies from 26 articles were included in this meta-analysis. The studies were divided into Caucasian (10 studies) [4, 22, 29–36], East Asian (5 studies) [23, 25, 37–39], South Asian (5 studies) [42–46] and Others (Arab (2 studies) [26, 27], Black African (3 studies) [22, 28, 29] and Brazilian (3 studies) [24, 40, 41]) subgroups. The collected data, performed with the R package meta in this meta-analysis, was displayed in Additional file 1: Table S2.

**Table 1:** The primary characteristics of the 28 studies

				T2DM			Control			
Study	Year	Population	Ethnicity	CC	CT	TT	CC	CT	TT	HWE
Ezzidi et al.	2009	Arabic Tunisian	Arab	250	396	217	181	235	95	0.227155
Saadi et al.	2008	Arab	Arab	30	54	11	71	94	23	0.388992
Humphries et al.	2006	Afro-Caribbean	Black African	141	136	30	161	124	26	0.75859
Yu et al.	2009	African American	Black African	255	212	48	1156	921	165	0.31807
Danquah et al.	2013	Ghanaian	Black African	273	323	78	182	165	28	0.257132
Yu et al.	2009	USA Caucasian	Caucasian	430	392	101	4295	3391	693	0.515248
Groves et al.	2006	English	Caucasian	771	960	270	1175	1084	217	0.944175
Humphries et al.	2006	European	Caucasian	601	665	193	1295	1001	197	0.854011
Cauchi et al.	2006	Austrian	Caucasian	200	208	78	555	432	88	0.759981
Dahlgren et al.	2007	Swedish	Caucasian	67	83	18	496	327	62	0.421344
Mayans et al.	2007	Swedish	Caucasian	452	318	54	532	253	35	0.480907
Van et al.	2007	Dutch	Caucasian	203	221	72	459	365	83	0.396927
Kimber et al.	2007	English	Caucasian	1405	1459	361	1714	1329	248	0.662991
De Silva et al.	2007	English	Caucasian	420	507	161	1032	887	180	0.58617
Vcelak et al.	2012	Czech	Caucasian	148	156	43	205	147	24	0.730572
Hayashi et al.	2007	Japanese	East Asian	1450	165	4	980	85	2	0.91209
Horikoshi et al.	2007	Japanese	East Asian	165	22	2	251	21	0	0.507848
Kazuaki et al.	2008	Japanese	East Asian	1921	228	5	1696	137	1	0.29539
Yasuharu et al.	2009	Japanese	East Asian	434	45	2	372	26	0	0.50056
Zheng et al.	2011	Chinese	East Asian	202	24	1	139	13	0	0.581813
Marquezine et al.	2007	Brazilian	Brazilian	45	54	13	564	603	128	0.070107
Barra et al.	2013	Brazilian	Brazilian	55	49	6	58	40	11	0.304112
Assmann et al.	2014	Brazilian	Brazilian	382	415	156	261	215	59	0.147418
Bodhini et al.	2007	Asian Indian	South Asian	462	455	114	555	391	92	0.531352
Chandak et al.	2007	Indian	South Asian	391	423	141	205	160	34	0.726021
Rees et al.	2008	UK South Asian	South Asian	352	360	116	222	166	44	0.12238
Gupta et al.	2010	Indian	South Asian	55	96	44	62	78	21	0.64658
Hussain et al.	2014	Indian	South Asian	25	36	7	43	35	4	0.349985

A total of 56628 participants (34,232 cases and 22,396 controls) of 28 studies from 26 articles were included in the study. The name of the first author, the publication year of, the population of the study, the ethnicity of the study, the genotypes of the case -control group and the *P* value of HWE. If the *p* value of HWE in control group met the selection criteria (*P* > 0.05), it would be preserved, otherwise the data would be removed

### Heterogeneity test

According to the genotypes shown in Table1, a total of 28 studies were analyzed by the dominant model, recessive model, additive model and allele model, respectively. The heterogeneity of all subgroups was shown in Table 2. According to the data displayed in Table 2, we didn’t get the significant heterogeneity in the dominant model (*p* = 0.39 and I^2^ = 5.00%), recessive model (*p* = 0.33 and I^2^ = 9%), additive model (CT vs CC: *p* = 0.76 and I^2^ = 0.00%), additive model (TT vs CC: *p* = 0.15 and I^2^ = 22%) and allele model (*p* = 0.08 and I^2^ = 29%). As the *p* value was more than 0.1, we selected the fixed effect model.

**Table 2:** The result of the heterogeneity in subgroup analyses

Subgroup	Dominant		Recessive		Additive(CT vs CC)		Allele		Additive(TT vs CC)	
	I^2^	P	I^2^	P	I^2^	P	I^2^	P	I^2^	P
Caucasian	28.00%	0.18	0.00%	0.51	9.00%	0.36	38.00%	0.1	20.00%	0.26
East Asian	0.00%	0.9	0.00%	0.85	0.00%	0.96	0.00%	0.82	0.00%	0.84
South Asian	0.00%	0.9	0.00%	0.47	0.00%	0.97	0.00%	0.7	0.00%	0.44
Others	0.00%	0.62	0.00%	0.19	0.00%	0.81	17.00%	0.29	29.00%	0.19
Total	5.00%	0.39	9.00%	0.33	0.00%	0.76	29.00%	0.08	22.00%	0.15

The I^2^ and *P* value were used to test the heterogeneity by the dominant model (TT+CT versus CC), recessive model (TT versus CC+CT), additive model (CT versus CC), additive model (TT versus CC) and allele model (T versus C), respectively

### Publication bias analysis and sensitivity analysis

**Fig. 2:**
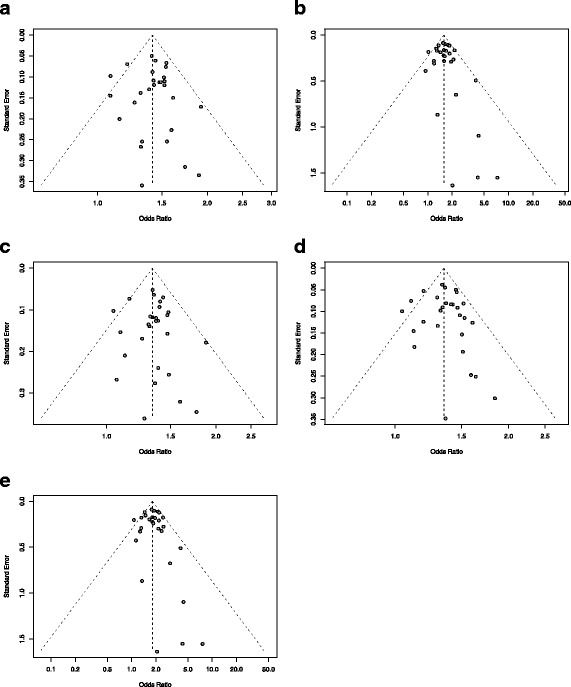
The funnel plots of publication bias in different models. The funnel plots showed the results of the publication bias analyses between rs7903146 and T2DM by using **a** Dominant Model, **b** Recessive Model, **c** Additive Model (CT vs CC), **d** Allele Model and **e** Additive Model (TT vs CC). The Y-axis indicated the standard error of each study, and the standard error was smaller, the effect of the meta-analysis would be better

The publication bias was not found in all models below. The *p* values of Begg’s test and Egger’s test for the dominant, recessive, additive (CT vs CC), additive (TT vs CC) and allele model are 0.7821 and 0.7352, 0.3635 and 0.441, 0.6354 and 0.711, 0.4528 and 0.5199, 0.6927 and 0.5673, respectively. The results were reflected in the funnel plots Fig. 2(a-e) directly.

### Association between rs7903146 and type 2 diabetes mellitus

**Fig. 3:**
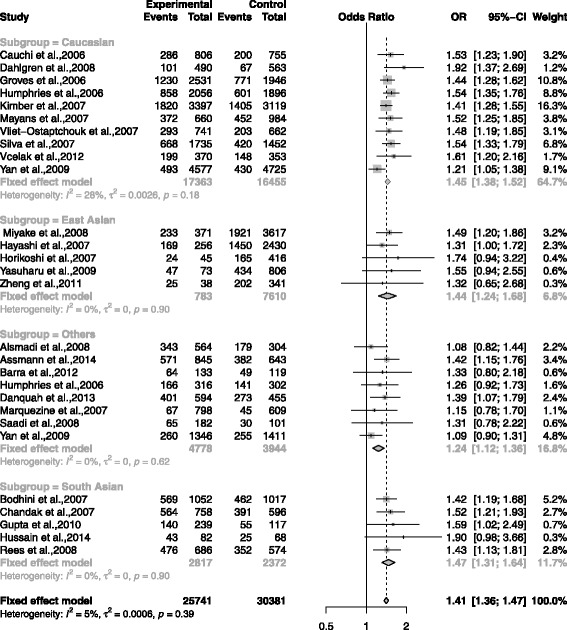
The forest plots for the meta-analysis of rs7903146 by using the dominant model. The data of CC/CT/TT was used in the dominant model (CT + TT vs CC)

**Fig. 4:**
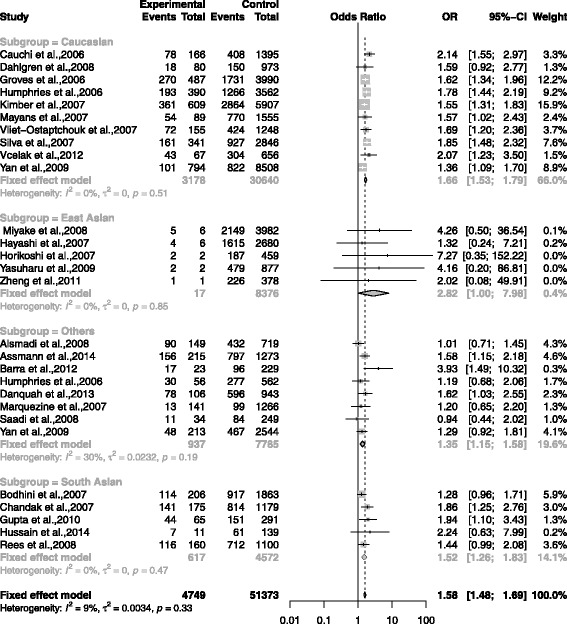
The forest plots for the meta-analysis of rs7903146 by using the recessive model. The data of CC/CT/TT was used in the recessive model (TT vs CC + CT)

**Fig. 5:**
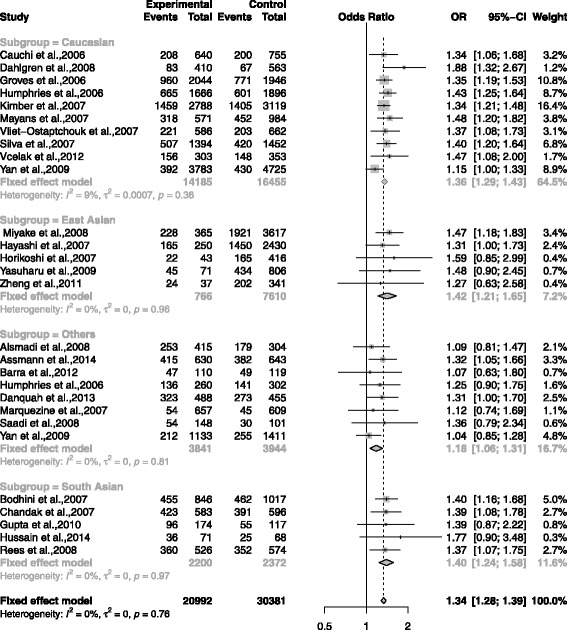
The forest plots for the meta-analysis of rs7903146 by using the additive model. The data of CC/CT/TT was used in the additive model (CT vs CC)

**Fig. 6:**
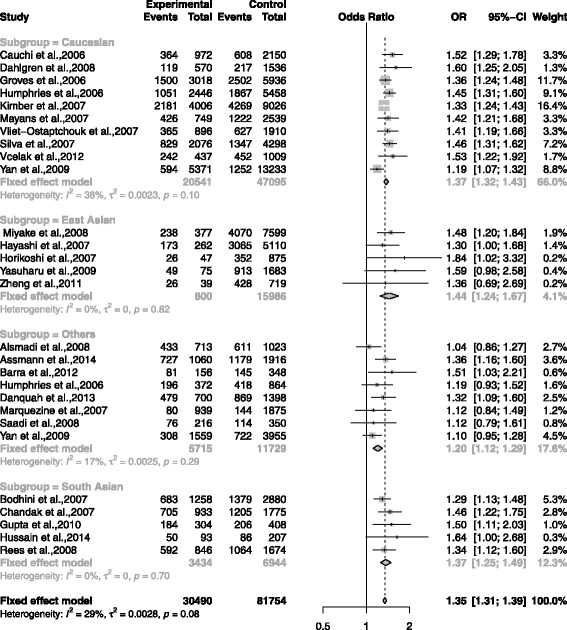
The forest plots for the meta-analysis of rs7903146 by using the allele model. The data of CC/CT/TT was used in the allele model (T vs C)

**Fig. 7:**
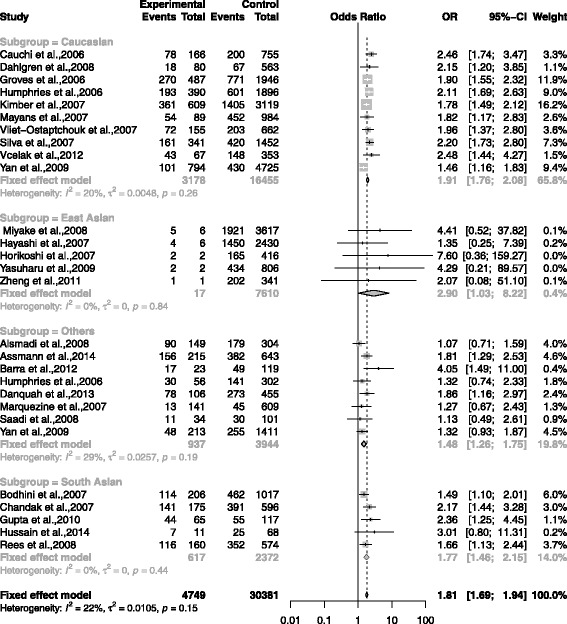
The forest plots for the meta-analysis of rs7903146 by using the additive model. The data of CC/CT/TT was used in the additive model (TT vs CC)

The association between rs7903146 and T2DM was shown in the forest plots: Figs. 3, 4, 5, 6 and 7 were the forest plots of the dominant model (TT+CT versus CC), recessive model (TT versus CC+CT), additive model (CT versus CC), allele model (T versus C) and additive model(TT versus CC), respectively. We made the Z test, and the result was displayed in the Table 3.

**Table 3:** The result of the Z test in subgroup analyses

Subgroup	Dominant		Recessive		Additive(CT vs CC)		Allele		Additive(TT vs CC)	
	Z	P	Z	P	Z	P	Z	P	Z	P
Caucasian	14.86	<0.0001	12.35	<0.0001	11.67	<0.0001	16.98	<0.0001	15.15	<0.0001
South Asian	4.69	<0.0001	1.95	0.0509	4.42	<0.0001	4.86	<0.0001	2.01	0.0446
East Asian	6.61	<0.0001	4.47	<0.0001	5.45	<0.0001	7.12	<0.0001	5.83	<0.0001
Others	4.17	<0.0001	3.75	0.0002	3.11	0.0019	4.89	<0.0001	4.65	<0.0001
Total	17.2	<0.0001	13.53	<0.0001	13.73	<0.0001	19.38	<0.0001	13.73	<0.0001

The Z test was performed with the dominant model (TT+CT versus CC), recessive model (TT versus CC+CT), additive model (CT versus CC), additive model (TT versus CC) and allele model (T versus C), respectively

In Caucasian subgroup, the results were shown as follows: dominant model (TT + CT vs CC): (OR = 1.45, CI = 1.38 - 1.52, *p* < 0.0001); recessive model (TT vs CC + CT): (OR = 1.66, CI = 1.53 - 1.79, *p* < 0.0001); additive model (CT vs CC): (OR = 1.36, CI = 1.29 - 1.43, *p* < 0.0001); additive model(TT vs CC): (OR = 1.91, CI = 1.76 - 2.08), *p* < 0.0001); allele model (T vs C): (OR = 1.37, CI = 1.32 - 1.43, *p* < 0.0001).

In East Asian subgroup, the results were shown as follows: dominant model (TT + CT vs CC): (OR = 1.44, CI = 1.24 - 1.68, *p* < 0.0001); recessive model (TT vs CC + CT): (OR = 2.82, CI = 1.00 - 7.98, *p* = 0.0509); additive model (CT vs CC): (OR = 1.42, CI = 1.21 - 1.65, *p*<0.0001); additive model(TT vs CC): (OR = 1.81, CI = 1.69 - 1.94, *p* < 0.0001); additive model(TT vs CC): (OR = 2.90, CI = 1.03 - 8.22, *p* = 0.0446); allele model (T vs C): (OR = 1.37, CI = 1.32 - 1.43, *p* < 0.0001).

In South Asian subgroup, the results were shown as follows: dominant model (TT + CT vs CC): (OR = 1.41, CI = 1.31 - 1.64, *p* < 0.0001); recessive model (TT vs CC + CT): (OR = 1.52, CI = 1.26 - 1.83, *p* < 0.0001); additive model (CT vs CC): (OR = 1.42, CI = 1.29 - 1.43, *p* < 0.0001); additive model(TT vs CC): (OR = 1.81, CI = 1.69 - 1.94, *p* < 0.0001); additive model(TT vs CC): (OR = 1.77, CI = 1.46 - 2.15, *p* < 0.0001) allele model (T vs C): (OR = 1.44, CI = 1.24 - 1.67, *p* < 0.0001).

In Others subgroup, the results were shown as follows: dominant model (TT + CT vs CC): (OR = 1.24, CI = 1.12 - 1.36, *p* < 0.0001); recessive model (TT vs CC + CT): (OR = 1.35, CI = 1.15 - 1.58, *p* = 0.0002); additive model (CT vs CC): (OR = 1.4, CI = 1.24 - 1.58, *p* = 0.0019); additive model(TT vs CC): (OR = 1.48, CI = 1.26 - 1.75, *p* < 0.0001); allele model (T vs C): (OR = 1.37, CI = 1.25 - 1.49, *p* < 0.0001).

In total groups, the results were shown as follows: dominant model (TT + CT vs CC): (OR = 1.41, CI = 1.36 - 1.47, *p* < 0.0001); recessive model (TT vs CC + CT): (OR = 1.58, CI = 1.48 - 1.69, *p* < 0.0001); additive model (CT vs CC): (OR = 1.34, CI = 1.28 - 1.39, *P* < 0.0001); additive model(TT vs CC): (OR = 1.81, CI = 1.69 - 1.94, *p* < 0.0001); allele model (T vs C): (OR = 1.35, CI = 1.31 - 1.39, *p* < 0.0001).

## Discussion

In the meta-analysis, 56628 participants (34232 cases and 22396 controls) of 28 studies from 26 articles were included. The result of the four subgroups (Caucasian, East Asian, South Asian and Others) suggested that rs7903146 was significantly associated with T2DM in all subgroups and the total groups.

We removed each one of the studies in the groups or any subgroups in the dominant, recessive, additive and allele model for testing the robustness of results, respectively. The results did not change significantly, which displayed that the conclusion was robust. The heterogeneity and publication bias were not found in our meta-analysis.

We used the keywords “rs7903146”, “type 2 diabetes” and “meta-analysis” to search in PubMed, and got nine articles [46, 56–63]. Our work was different from others. We analyzed the association between rs7903146 and T2DM in Caucasian, East Asian, South Asian and Others groups. We did not find a significant heterogeneity in all subgroup analyses, so the fixed effect model was used. We found that rs7903146 was associated with T2DM in Caucasian, East Asian, South Asian and other ethnicities significantly.

Some limitations existed in this meta-analysis. Firstly, considering the heterogeneity in all subgroup analyses, we excluded 9 articles. More articles should be added into the meta-analysis. Secondly, some of the same cases or controls may be used in different studies.

## Conclusion

The meta-analysis suggested that rs7903146 was significantly associated with T2DM in Caucasian, East Asian, South Asian and other ethnicities.

## Additional file

Additional file 1 Table S1. The detailed information about the search strategy. Table S2. The collected data in the meta-analysis. (XLSX 13 kb)

## Abbreviations

CIs: Confidence intervals
HWE: Hardy-Weinberg Equilibrium
ORs: Odds ratio
SNP: Single necluotide polymorphism
T2DM: Type 2 diabetes mellitus
TCF7L2: Transcription factor 7-like 2
